# Supplementary material for: DMRforPairs: identifying Differentially Methylated Regions between unique samples using array based methylation profiles
Source: BMC Bioinformatics. 2014 May 15;15:141. doi: 10.1186/1471-2105-15-141 (PMC4046028; doi:10.1186/1471-2105-15-141)
Supplement: Additional file 2 — DMRforPairs output for the comparison of A431-MCF7 and NA17018-NA17105. Please start from the HTML files in each folder. Available via the BMC Bioinformatics website. [file 1471-2105-15-141-S2.zip › 1394847754114233_MOESM2_ESM/A431_MCF7/figures/33940.pdf]

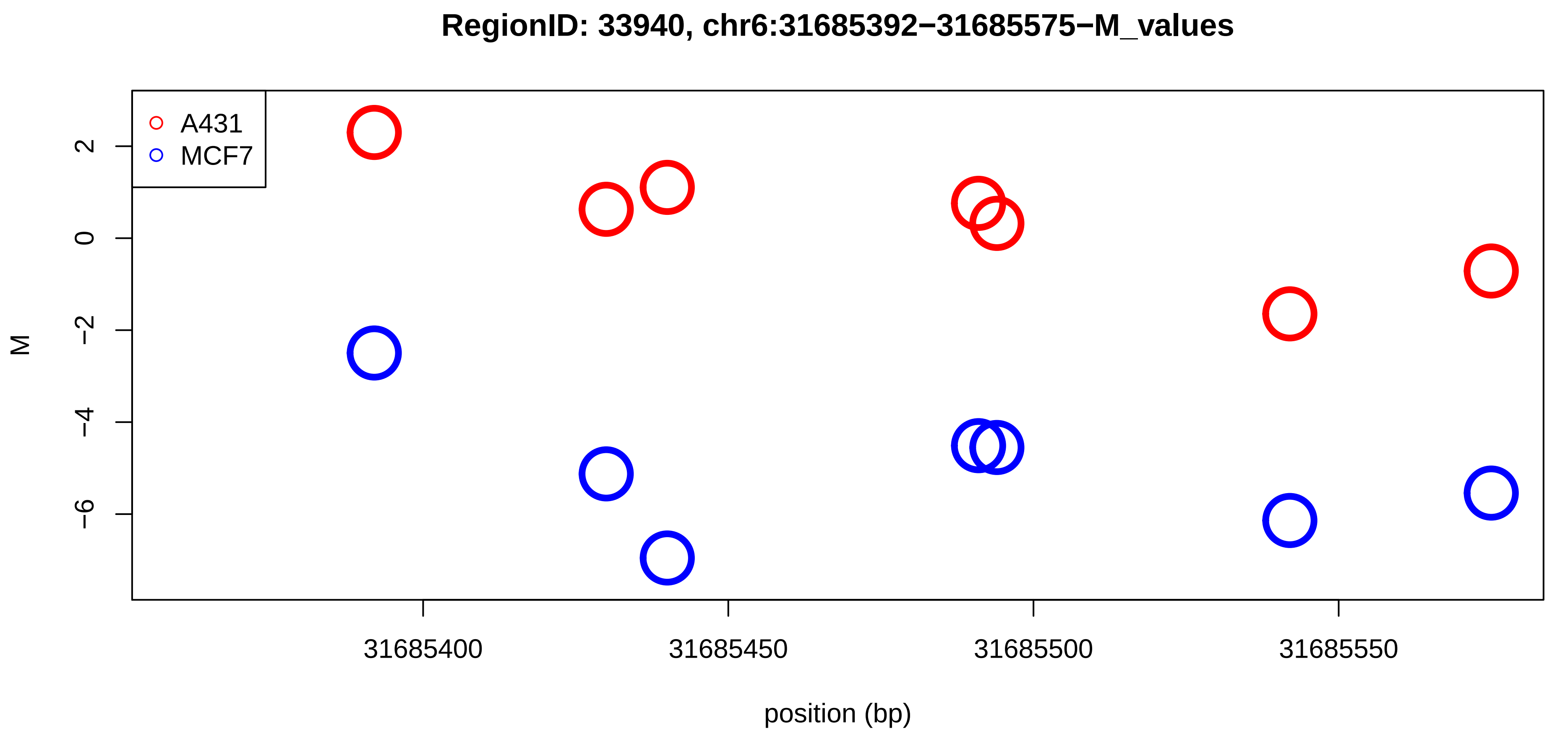
RegionID: 33940, chr6:31685392−31685575−M_values
A431
MCF7
M
2
0
−2
−4
−6
31685400
31685450
31685500
31685550
position (bp)

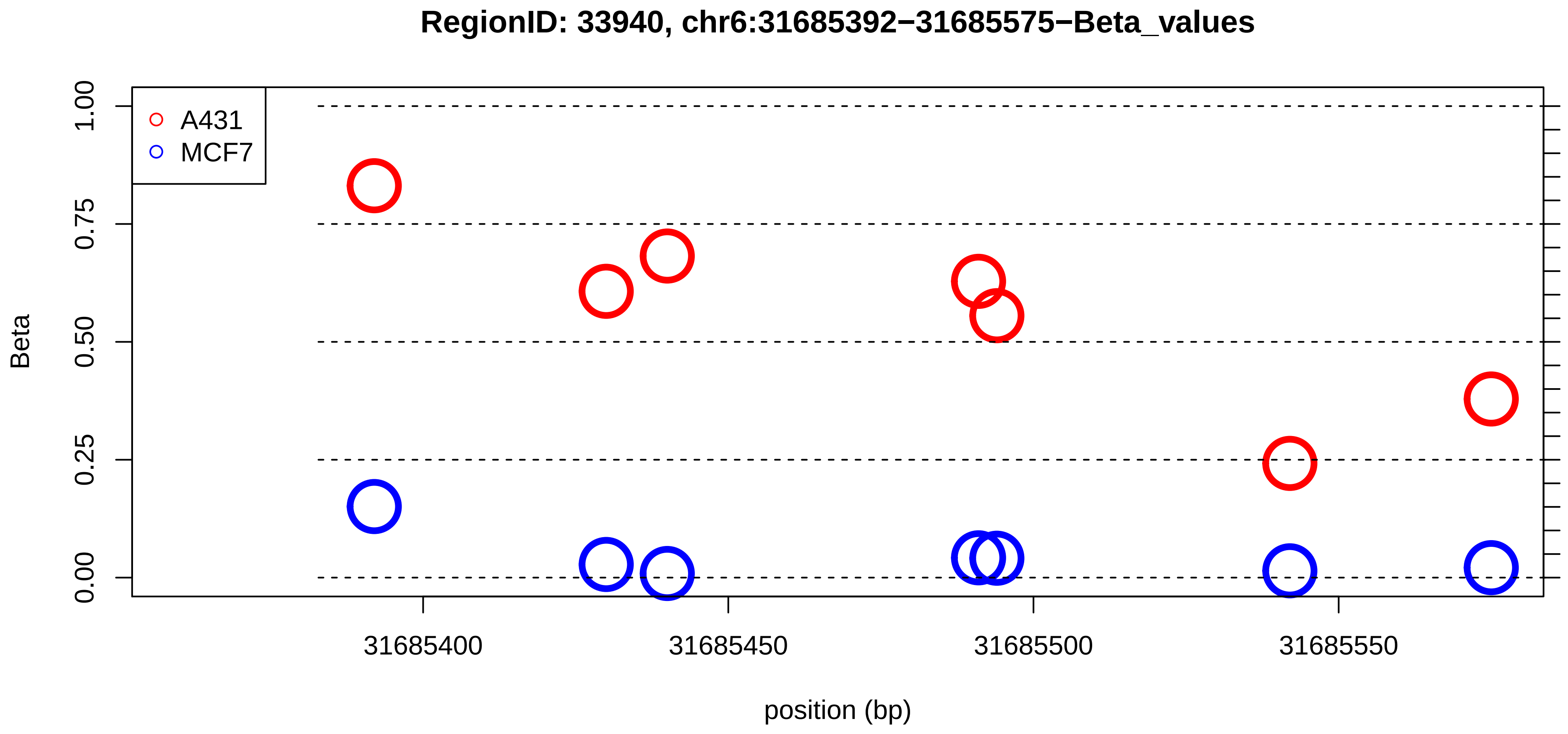
RegionID: 33940, chr6:31685392−31685575−Beta_values
A431
MCF7
Beta
1.00
0.75
0.50
0.25
0.00
31685400
31685450
31685500
31685550
position (bp)

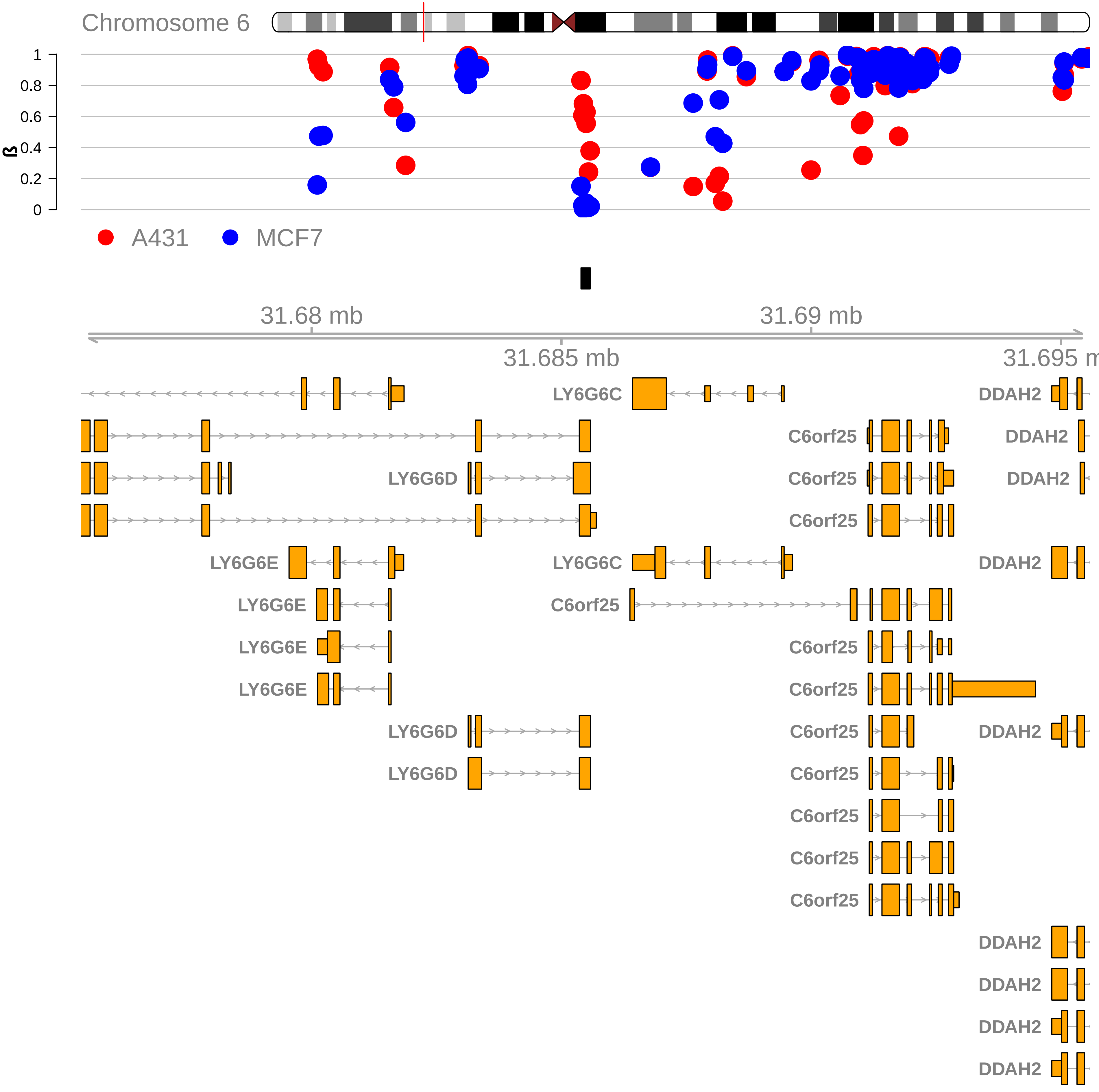

Chromosome 6
ß
1
0.8
0.6
0.4
0.2
0
A431
MCF7
31.68 mb
31.69 mb
31.685 mb
31.695 mb
LY6G6C
DDAH2
C6orf25
LY6G6D
LY6G6E
